# Trends in Inpatient Psychiatry Referrals: A Retrospective Study of Consultation-Liaison Psychiatry Services

**DOI:** 10.7759/cureus.108207

**Published:** 2026-05-03

**Authors:** Kritika Agarwal, Tanu Kumari, Sulagna Mallik, Pankaj Kumar, Himel Mondal

**Affiliations:** 1 Psychiatry, Apollo Excelcare Hospital, Guwahati, IND; 2 Psychiatry, Narayan Medical College and Hospital, Sasaram, IND; 3 Geriatric Psychiatry, National Institute of Mental Health and Neurosciences, Bengaluru, IND; 4 Psychiatry, All India Institute of Medical Sciences, Patna, Patna, IND; 5 Physiology, All India Institute of Medical Sciences, Deoghar, Deoghar, IND

**Keywords:** comorbidity, consultation-liaison psychiatry, covid-19, delirium, hospital psychiatry, inpatients, mental health services, psychiatry referrals, retrospective study, socio-demographic factors

## Abstract

Background

The coronavirus disease 2019 (COVID-19) pandemic has significantly impacted healthcare delivery, including consultation-liaison psychiatry (CLP) services, by altering patient profiles and referral dynamics. Understanding these changes is crucial for optimizing mental health care within general hospital settings. This study aimed to compare the pattern of CLP referrals among inpatients during the pre-COVID and post-COVID periods.

Methods

A retrospective chart review was conducted at the All India Institute of Medical Sciences, Patna, India. Data from inpatients referred to the CLP team during March-August 2019 (pre-COVID) and March-August 2022 (post-COVID) were analyzed. Six months was selected because the number of referrals to CLP is relatively low, and a longer duration was required to obtain a meaningful sample size for analysis. Accordingly, we included approximately six months of data from the pre-COVID period and used a comparable timeframe from another year during the pandemic to ensure consistency and allow for valid comparison. Socio-demographic variables, referral sources, psychiatric diagnoses, family history, and comorbid physical illnesses were compared using the chi-square test.

Results

A total of 285 patients were included in the study. The number of patients seen by the CLP team increased from 107 in the pre-COVID period to 178 in the post-COVID period, representing a 66.4% increase. There was a significant shift in age distribution (p-value = 0.011), with a decrease in patients aged >60 years in the post-COVID period (56 (52.3%) to 57 (32%), p-value = 0.011). Referral patterns shifted, with increased referrals from emergency (7 (6.5%) to 23 (12.9%)) and general medicine (13 (12.1%) to 39 (21.9%)), and decreased referrals from accidents and trauma (16 (15%) to 7 (3.9%), p-value = 0.007). Delirium showed a substantial rise (8 (7.5%) to 54 (30.3%)), while mood disorders (14 (13.1%) to 13 (7.3%)) and psychotic disorders (16 (15%) to 16 (9%)) declined (p-value = 0.0006). Family history of psychiatric illness remained largely unchanged.

Conclusion

The study demonstrates important shifts in psychiatry referral patterns and patient characteristics following the COVID-19 pandemic. These findings highlight the need for flexible and responsive CLP services within hospital settings. They also provide useful insights for planning mental health services and strengthening preparedness for future public health emergencies.

## Introduction

Consultation-liaison psychiatry (CLP) is a relatively young subspecialty of psychiatry [1]. Liaison psychiatry, also known as consultative psychiatry or CLP, focuses on the interface between psychiatry and other medical and surgical specialties [2]. The term CLP is often used interchangeably with “psychosomatic medicine,” which refers to a discipline concerned with understanding the interplay of biological and psychosocial factors in the development, course, and outcomes of diseases [3].

Within this interface, psychiatric comorbidities are highly prevalent among hospitalized patients, with conditions such as delirium, depression, anxiety disorders, substance use disorders, and adjustment disorders frequently encountered [4]. These comorbidities are associated with prolonged hospital stays, increased healthcare costs, poorer treatment adherence, and higher morbidity and mortality [5]. Notably, the pattern of consultation-liaison referrals to psychiatry has remained relatively stable in recent years, with alcohol-related disorders, delirium, and adjustment disorders being the most frequent diagnoses [6-8].

However, this relatively stable pattern has been challenged by the coronavirus disease 2019 (COVID-19) pandemic, which has been associated with a notable increase in psychiatric illnesses across both the general population and affected patients [9]. Additionally, individuals infected with the virus have shown a higher risk of developing neuropsychiatric complications, including delirium and cognitive disturbances, particularly in severe cases [10]. The pandemic has also contributed to the exacerbation of pre-existing mental health conditions and created barriers to accessing care, further amplifying the overall psychiatric burden [11].

Given these shifts, understanding the changing trends in psychiatry referrals has become essential for optimizing service delivery, resource allocation, and training of healthcare professionals. Interestingly, the COVID-19 pandemic was associated with a marked reduction in primary care-recorded mental illness and service utilization, despite evidence of worsening population mental health [12]. In this context, the present study aims to evaluate trends in psychiatry referrals for inpatients assessed by CLP through a retrospective chart review. By examining patterns over time, this study seeks to provide insights into evolving clinical practices and inform strategies to enhance the integration of mental health care within general hospital settings.

## Materials and methods

This study was a retrospective chart review conducted at the All India Institute of Medical Sciences, Patna, India. The study included all inpatients referred to and assessed by the CLP team during two distinct time periods: from March 1, 2019, to August 31, 2019 (pre-COVID period) and from March 1, 2022, to August 31, 2022 (post-COVID period). Six months was selected because the number of referrals to CLP is relatively low, and a longer duration was required to obtain a meaningful sample size for analysis. Additionally, a formal pre-study sample size calculation was not feasible due to the limited availability of prior data on referral rates in this setting. Using the same calendar months helps control for seasonal variations in hospital admissions and psychiatric referrals, ensuring that observed differences are less likely to be due to temporal fluctuations.

Data were obtained from CLP service records using a semi-structured proforma routinely maintained by the team (available in Appendix 1). The proforma captured relevant sociodemographic and clinical variables, including age, gender, religion, occupation, marital status, referring department, medical comorbidities, psychiatric diagnosis, and significant past or family history. All eligible inpatient referrals during the specified periods were included; eligibility was defined as any inpatient formally referred to the CLP team for psychiatric evaluation by the treating medical or surgical unit during the study period. Data were collected by KA and TK, both of whom are trained psychiatrists with more than two years of clinical experience at the time of data collection. To reduce potential bias, data extraction was performed using a standardized data collection form, and clear operational definitions were applied for all variables. Psychiatric diagnoses were made in accordance with ICD-10 criteria [13]. Any discrepancies were resolved through discussion and consensus, ensuring consistency and reliability in the collected data.

Categorical variables were summarized as frequencies and percentages. Comparisons between the two groups for qualitative variables were performed using the chi-square test or Fisher’s exact test, as appropriate. Fisher’s exact test with Monte Carlo simulation was used for a large contingency table. A p-value of less than 0.05 was considered statistically significant. Data analysis was performed using GraphPad Prism 9.5.0 (GraphPad Software, Inc., San Diego, CA, USA).

## Results

The number of patients seen by the CLP team increased from 107 in the pre-COVID-19 period to 178 in the post-COVID period, representing a 66.4% increase, calculated as the relative change from the pre-COVID baseline $\left( \frac{178 - 107}{107} \right) \times 100$. There was a significant shift in age distribution post-COVID (p-value = 0.011), with a decrease in elderly patients (>60 years) and an increase in adults aged 18-40 years. Gender distribution remained comparable between periods. Religion and marital status showed significant differences (p-value = 0.003 and p-value = 0.005, respectively). Detailed demographic profiles of the patients are shown in Table 1.

**Table 1:** Comparison of the pattern of socio-demographic variables among inpatients for whom psychiatry referrals were received during the pre-COVID and post-COVID periods The p-values are from Fisher’s exact test; n denotes number; *p-value from the chi-square test.

Parameter	Category	Pre-COVID (n = 107)	Post-COVID (n = 178)	Total (n = 285)	p-values
		n (%)			
Age (years)	≥12	1 (0.93%)	5 (2.8%)	6 (2.1%)	0.011
	13 to 17	0	2 (1.1%)	2 (0.7%)	
	18 to 40	28 (26.2%)	64 (36%)	92 (32.3%)	
	41 to 60	22 (20.6%)	50 (28.1%)	72 (25.3%)	
	>60	56 (52.3%)	57 (32%)	113 (39.6%)	
Gender	Female	58 (54.2%)	79 (44.4%)	137 (48.1%)	0.14*
	Male	49 (45.8%)	99 (55.6%)	148 (51.9%)	
Religion	Hindu	83 (77.6%)	159 (89.3%)	242 (84.9%)	0.003
	Muslim	15 (14%)	17 (9.6%)	32 (11.2%)	
	Others	9 (8.4%)	2 (1.1%)	11 (3.9%)	
Marital status	Unknown	0	2 (1.1%)	2 (0.7%)	0.005
	Married	58 (54.2%)	126 (70.8%)	184 (64.6%)	
	Unmarried	49 (45.78%)	50 (28.1%)	99 (34.7%)	
Occupation	Unemployed	29 (27.1%)	102 (57.1%)	131 (45.9%)	<0.0001
	Student	31 (29%)	7 (3.9%)	38 (13.3%)	
	Housewife	30 (28%)	17 (9.6%)	47 (16.5%)	
	Self employed	9 (8.4%)	22 (12.4%)	31 (10.9%)	
	Private employed	6 (5.6%)	19 (10.7%)	25 (8.8%)	
	Government employed	2 (1.9%)	9 (5.1%)	11 (3.9%)	
	Retired	0	2 (1.1%)	2 (0.7%)	
Education	Illiterate	18 (16.8%)	29 (16.3%)	47 (16.5%)	<0.0001
	Primary school	32 (29.9%)	40 (22.5%)	72 (25.3%)	
	Middle school	27 (25.2%)	46 (25.8%)	73 (25.6%)	
	High school	4 (3.7%)	47 (26.4%)	51 (17.9%)	
	Intermediate	14 (13.1)	16 (9%)	30 (10.5%)	
	Graduate	11 (10.3%)	0	11 (3.86%)	
	Post graduate	1 (0.9%)	0	1 (0.4%)	

Referrals to psychiatry were most commonly received from the super specialty medicine departments in the pre-COVID period. Post-COVID, there was a noticeable increase in referrals from emergency and general medicine, while referrals from accidents and trauma and otorhinolaryngology decreased. Department-wise referral percentages are shown in Table 2.

**Table 2:** Comparison of the pattern of psychiatry referrals of inpatients coming from different departments during the pre-COVID and post-COVID periods p-value from Fisher’s exact test with Monte Carlo simulation = 0.007; n denotes number.

Departments	Pre-COVID (n = 107)	Post-COVID (n = 178)	Total (n = 285)
	n (%)		
Dental	1 (0.9%)	0	1 (0.4%)
Dermatology	4 (3.7%)	7 (3.9%)	11 (3.9%)
Emergency	7 (6.5%)	23 (12.9%)	30 (10.4%)
Accidents and trauma	16 (15%)	7 (3.9%)	23 (8.1%)
Otorhinolaryngology	7 (6.5%)	5 (2.8%)	12 (4.2%)
General medicine	13 (12.1%)	39 (21.9%)	52 (18.2%)
General surgery	5 (4.7%)	10 (5.6%)	15 (5.3%)
Obstetrics and gynecology	3 (2.8%)	7 (3.9%)	10 (3.5%)
Intensive care unit	7 (6.5%)	8 (4.5%)	15 (5.3%)
Ophthalmology	1 (0.9%)	0	1 (0.4%)
Orthopedics	4 (3.7%)	14 (7.9%)	18 (6.3%)
Paediatrics	10 (9.3%)	15 (8.4%)	25 (8.8%)
Radiotherapy	1 (0.9%)	2 (1.1%)	3 (1.1%)
Super specialty medicine departments	19 (17.8%)	18 (10.2%)	37 (13%)
Super specialty surgery departments	9 (8.4%)	23 (13%)	32 (11.2%)

Table 3 shows the family history of psychiatric diagnoses. The majority of patients in both periods had no family history of psychiatric illness.

**Table 3:** Comparison of family history of psychiatric diagnosis among inpatients for whom psychiatric referrals were received during the pre-COVID and post-COVID periods Chi-square value = 1.75; degrees of freedom = 1; p-value = 0.185; n denotes number.

Family history of psychiatric diagnosis	Pre-COVID (n = 107)	Post-COVID (n = 178)	Total (n = 285)
	n (%)		
Absent	98 (91.6%)	171 (96.1%)	269 (94.4%)
Present	9 (8.4%)	7 (3.9%)	16 (5.6%)

Neurotic, stress-related, and somatoform disorders were the most common diagnoses in both periods, followed by delirium and psychotic spectrum disorders. There was a marked increase in delirium cases post-COVID, while mood and psychotic disorders showed a relative decline. The proportion of patients with no psychiatric diagnosis remained comparable across both periods (Table 4).

**Table 4:** Comparison of psychiatric diagnoses among inpatients for whom psychiatry referrals were received during the pre-COVID and post-COVID periods p-value from Fisher’s exact test with Monte Carlo simulation = 0.0006; n denotes number.

Psychiatric diagnosis	Pre-COVID (n = 107)	Post-COVID (n = 178)	Total (n = 285)
	n (%)		
Behavioral syndrome associated with physiological disturbance	5 (4.7%)	7 (3.9%)	12 (4.2%)
Catatonia	4 (3.7%)	3 (1.7%)	7 (2.5%)
Delirium	8 (7.5%)	54 (30.3%)	62 (21.8%)
Mood disorders	14 (13.1%)	13 (7.3%)	27 (9.5%)
Neurotic, stress-related, and somatoform disorders	30 (28%)	45 (25.3%)	75 (26.3%)
Personality disorder	2 (1.9%)	4 (2.2%)	6 (2.1%)
Psychoactive substance use disorder	4 (3.7%)	4 (2.2%)	8 (2.8%)
Psychological development disorder	2 (1.9%)	0	2 (0.7%)
Psychotic spectrum disorders	16 (15%)	16 (9%)	32 (11.2%)
No psychiatric diagnosis	22 (20.6%)	32 (18%)	54 (18.9%)

As presented in Table 5, medical illnesses were the predominant comorbid conditions in both periods, with a slight decline post-COVID. At the same time, surgical comorbidities showed a modest increase, indicating a shift in the pattern of physical illnesses among referred patients.

**Table 5:** Comparison of comorbid physical illness among inpatients for whom psychiatry referrals were received during the pre-COVID and post-COVID periods Chi-square = 0.21; p-value = 0.64; n denotes number.

Co-morbid physical illness	Pre-COVID (n = 107)	Post-COVID (n = 178)	Total (n = 285)
	n (%)		
Surgical illness	33 (30.8%)	61 (34.3%)	94 (33%)
Medical illness	74 (69.2%)	117 (65.7%)	191 (67%)

## Discussion

In our study, the number of patients seen by the CLP team increased from the pre-COVID (2019) to the post-COVID (2022) period, rising from 107 to 178 patients, representing a 66% increase. The marked increase in delirium in the post-COVID period is likely due to the direct neuropsychiatric effects of COVID-19, higher severity of illness, and increased ICU admissions. Improved awareness and screening for delirium during the pandemic might have also increased its detection. A study by Lele et al. from Australia reported an approximately 25% rise in CLP referrals during the COVID-19 pandemic, with a delayed but sustained increase even after infection rates declined [14]. Furthermore, El Hayek et al. also concluded that the need for CLP significantly increased in COVID-19 wards during the pandemic, with common diagnoses including delirium, depression, and anxiety [15]. In contrast, Grover et al. from India reported a 44% reduction in overall referrals to CLP; however, there was a relative increase in referrals for self-harm, abnormal behavior, and depressive disorders [16]. The discordance with our study may be explained by differences in hospital functioning and patient flow during the COVID-19 pandemic, where some centers experienced reduced admissions and restricted referrals. In contrast, our setting may have seen increased service demand, possibly due to differences in referral practices, case mix, and availability of CLP services. 

A relative increase in referrals from emergency services and general medicine suggests a growing recognition of acute psychiatric needs in patients presenting with medical illnesses, possibly reflecting heightened psychological distress and neuropsychiatric manifestations associated with the pandemic [17]. Referrals from departments such as accidents and trauma showed a relative decline. Lockdowns, travel restrictions, and reduced outdoor activity led to a decline in road traffic accidents and trauma-related admissions [18], thereby decreasing the pool of patients requiring psychiatric evaluation. Additionally, the reorganization of emergency services and prioritization of COVID-related care may have limited routine psychiatric referrals in acute trauma settings [19].

This study remains important even after the COVID-19 pandemic has subsided, as it provides valuable insights into how patterns of psychiatry referrals and patient profiles can shift during large-scale health crises. Understanding these changes can aid future preparedness by helping healthcare systems anticipate the mental health needs of patients during similar pandemics or emergencies. The findings can guide the development of more responsive CLP services, improve interdisciplinary collaboration, and support better resource allocation. Additionally, it contributes to the existing body of evidence on the long-term impact of pandemics on mental health care delivery [20].

The study has some limitations. It was conducted at a single tertiary care center, which may limit the generalizability of the findings to other settings. The retrospective chart review design may be associated with incomplete or missing data, potentially affecting the accuracy of results, and inherently carries a risk of selection and information bias. Although efforts were made to minimize this through the use of a standardized proforma and consensus-based data extraction, residual bias cannot be excluded. The sample size was not determined through a priori statistical calculation due to the retrospective nature of the study, and all eligible cases within the defined time periods were included; therefore, the study may be underpowered to detect smaller differences. Additionally, specific diagnostic subcategories were not predefined at the time of data collection, which may have limited the granularity of classification in some groups. The comparison between two specific time periods rather than continuous data across years may not fully capture temporal trends. Potential confounding factors, such as severity of illness, changes in hospital policies, and variations in referral practices during the pandemic, were not controlled for. Additionally, long-term patient outcomes were not assessed, limiting understanding of the sustained impact of the observed changes.

## Conclusions

The study highlights a clear change in the pattern of psychiatry referrals between the pre- and post-COVID periods, reflecting the wider impact of the pandemic on healthcare utilization and mental health needs. Variations were noted in patient profiles, referral pathways, and the spectrum of psychiatric conditions encountered. These observations underscore the evolving demands on CLP services and emphasize the need for adaptive planning and preparedness in future healthcare crises.

## Appendices

Appendix 1

**Table 6:** The proforma used to collect data from the registry of the institution CLP: Consultation-liaison psychiatry, COVID: Coronavirus disease 2019, ID: Identification, ICD: International classification of diseases, DD: Day, MM: Month, YYYY: Year

Section	Variable	Question or item	Response options
Identification details	Patient ID	Unique patient identification number	Alphanumeric
	Date of referral	Date when referral was made	DD/MM/YYYY
	Study period	Period of admission/referral	Pre-COVID (Mar-Aug 2019)
			Post-COVID (Mar-Aug 2022)
Sociodemographic details	Age	Age of the patient	In years
	Gender	Sex of the patient	Male
			Female
			Others
	Religion	Religion of the patient	Hindu
			Muslim
			Others
	Marital status	Current marital status	Married
			Unmarried
			Others
	Occupation	Current occupation	Unemployed
			Student
			Housewife
			Self-employed
			Private employed
			Government employed
			Retired
	Education	Highest level of education attained	Illiterate
			Primary
			Middle
			High school
			Intermediate
			Graduate
			Postgraduate
Clinical referral details	Referring department	Department from which referral was made	Dental
			Dermatology
			Emergency
			Trauma
			Otorhinolaryngology
			General medicine
			General surgery
			Obstetrics and gynecology
			Intensive care unit
			Ophthalmology
			Orthopedics
			Pediatrics
			Radiotherapy
			Super-specialty medicine
			Super-specialty surgery
Clinical variables	Presenting complaints	Reason for psychiatric referral	Free text
	Psychiatric diagnosis	Final diagnosis by CLP team	As per ICD classification (specify)
	Diagnostic category	Broad category of psychiatric disorders	Delirium
			Mood disorder
			Neurotic & stress-related
			Substance use
			Personality disorder
			-
Past and family history	Past psychiatric history	History of past psychiatric illness	Present
			Absent
	Family history	Family history of psychiatric illness	Present
			Absent
Comorbid physical illness	Type of comorbidity	Nature of physical illness	Medical
			Surgical
	Details of illness	Specific diagnosis	Free text
Additional information	Treatment given	Psychiatric intervention provided	Medication
			Counseling
			Both
	Outcome (if available)	Clinical outcome during admission	Improved
			Same
			Deteriorated
			Not recorded
